# Drive-By-Wire Development Process Based on ROS for an Autonomous Electric Vehicle

**DOI:** 10.3390/s20216121

**Published:** 2020-10-27

**Authors:** J. Felipe Arango, Luis M. Bergasa, Pedro A. Revenga, Rafael Barea, Elena López-Guillén, Carlos Gómez-Huélamo, Javier Araluce, Rodrigo Gutiérrez

**Affiliations:** Electronics Department, University of Alcalá, Campus Universitario, 28805 Alcalá de Henares, Spain; juanfelipe.arango@edu.uah.es (J.F.A.); pedro.revenga@uah.es (P.A.R.); rafael.barea@uah.es (R.B.); elena.lopezg@uah.es (E.L.-G); carlos.gomezh@edu.uah.es (C.G.H.); javier.araluce@edu.uah.es (J.A.); rodrigo.gutierrez@edu.uah.es (R.G.)

**Keywords:** drive-by-wire, steer-by-wire, throttle-by-wire, autonomous vehicle, robot operating system (ROS), automotive electronics, electric actuators

## Abstract

This paper presents the development process of a robust and ROS-based Drive-By-Wire system designed for an autonomous electric vehicle from scratch over an open source chassis. A revision of the vehicle characteristics and the different modules of our navigation architecture is carried out to put in context our Drive-by-Wire system. The system is composed of a Steer-By-Wire module and a Throttle-By-Wire module that allow driving the vehicle by using some commands of lineal speed and curvature, which are sent through a local network from the control unit of the vehicle. Additionally, a Manual/Automatic switching system has been implemented, which allows the driver to activate the autonomous driving and safely taking control of the vehicle at any time. Finally, some validation tests were performed for our Drive-By-Wire system, as a part of our whole autonomous navigation architecture, showing the good working of our proposal. The results prove that the Drive-By-Wire system has the behaviour and necessary requirements to automate an electric vehicle. In addition, after 812 h of testing, it was proven that it is a robust Drive-By-Wire system, with high reliability. The developed system is the basis for the validation and implementation of new autonomous navigation techniques developed within the group in a real vehicle.

## 1. Introduction

The high demand for mobility, the progress of the automation and the concern for the demotion of the environment has caused that in the last decades the technological advances in the automotive industry have exponentially grown. The main objective of these advances is to develop smarter vehicles that make transport safer and more efficient. Nowadays, the most challenging for smart vehicles is autonomous driving. Even though it could seem a trivial task for humans, actually it involves multiple sub-tasks difficult to manage for a machine in real time. These sub-tasks can be grouped in different topics: environment perception, localisation, planning, vehicle control and decision-making [1]. The upper-level control is not possible without a low-level control system sufficiently accurate and robust.

To really validate autonomous vehicle navigation techniques, it is necessary to dispose of an automated vehicle which allows sending control commands through an interface known as Drive-By-Wire.

In the Techs4AgeCar project developed at the University of Alcalá, the objective is implementing a self-driving open-source electric car, cheap, ecologic and adapted to elderly people. After a revision of the state of art, we decided to build our own car, covering all the design phases involved in this process: mechanic, electronic, perception, planning, control, navigation and validation in simulation as well as in a real prototype [2].

The project is based on ROS (Robot Operating System), an open-source framework that provide libraries and tools for helping the software developers to create robot applications [3].

In this paper, we present the development process of the Drive-By-Wire system designed for our autonomous vehicle prototype.

The remaining paper is arranged as follows. Section 2 presents a bibliographic review of the Drive-By-Wire technology. Section 3 introduces a general view of the Techs4AgeCar’s project, where the vehicle and each of the modules of the navigation architecture are briefly explained. Section 4 explains the Drive-By-Wire system’s design. Finally, Section 5 and Section 6 expose the results and conclusions, respectively.

## 2. Related Work

In 1955, the company Lucas Industries designed the first transistorised ignition system for a vehicle. The transistor made its entry in the automobile, and with it the electronic age on the automotive industry started.

Some years later, ADAS (Advanced Driver Assistance Systems) began to be developed. ADAS includes all the electronic devices that assist the driver and the passengers, either actively or passively. The purpose of these systems is to guarantee safety and comfort for driving [4].

In 1965, Ford’s Mercury Park Lane model with EHPS (Electro-Hydraulic Powered Steering) was introduced. By 1968, Volkswagen launched the first electronic fuel injection system-based vehicle. In the late 1970s, Mercedes released ABS (Antilock Braking System). Moreover, in 1990, Honda introduced the NSX model with EPS (Electrical Powered Steering) system [5]. Due to the high cost, these electronic systems were only incorporated into top luxury vehicles at the beginning. Since the awakening of ADAS, the automotive industry has not stopped conducting exhaustive research and development in terms of electronic modules, regarding efficiency and reliability, which has allowed incorporating them in most current vehicles.

At the beginning of the current decade, a new paradigm emerged in the automotive industry called Drive-By-Wire. The scope of this new concept is to develop a reliable communication between the control elements and the vehicle’s actuators by wire, removing most common mechanical elements. According to the state of the art, there are three types of Drive-By-Wire systems: Steer-By-Wire, Throttle-By-Wire and Brake-By-Wire.

In 1988, the first Throttle-By-Wire system appeared, integrated by the BMW 7 series, which had an acceleration control system called EML (Elektronische Motorleistungsregelung) [6]. By 2002, Toyota launched the Estima Hybrid model in Japan, in which the first Brake-By-Wire system, called ECB (Electronically Controlled Brake), was developed [7].

Finally, the first automobile manufacturer to announce the incorporation of a Steer-By-Wire system was Nissan, with the 2013 Infiniti Q50 model and the new technology called DAS (Direct Adaptive Steering) [8]. Since this moment, this new technology started to be incorporated into the new models of all their vehicles.

All explained technologies have been developed for commercial vehicles. Nevertheless, due to patented licenses associated to these emerged technologies, most automobile manufacturers do not open their systems to third parties. Then, if an external designer wants to use this functionality, he is obliged to develop external actuators to control the steering wheel, accelerator and brake. For example, in 1997, the company AB Dynamics developed the SR30 [9], the first Steer-By-Wire robot, which was used by Goodyear to conduct driving tests.

The implementation of these systems and the increasing efficiency and reliability of electronic applications in vehicles have given rise to new challenges for the automotive industry. One of the main challenges, and probably the most important, has been the transmission of data among the different control units.

Regarding data transmission, the CAN-Bus (Controller Area Network) communications protocol was developed by the German company Bosch in 1982 [10]. Its implementation in vehicles has shown great progress, helping to simplify communications and the distribution of tasks among units. In addition to this communications protocol, other approaches were developed in the automotive sector, such as the LIN-Bus (Local Interconnect Network) and the TTCAN (Time Triggered CAN) [11]. Nevertheless, their use did not have as much prominence as the CAN protocol. In that sense, FlexRay [12] is a new communications protocol for automotive data buses, developed by the FlexRay Consortium between 2000 and 2009. It is widely considered as a more advanced protocol than CAN in terms of cost and performance. The first vehicle on the market that added this technology was the BMW X5, launched on the market in 2007.

### 2.1. Steer-By-Wire Technology

The EPS (Electrical Powered Steering) incorporated in current vehicles can be understood as an intermediate link between the mechanical steering column and the Steer-By-Wire system, since the driver continues to provide some torque to the mechanism.

On the other hand, the Steer-By-Wire system uses the measurement of a position sensor on the steering wheel and information from the vehicle’s sensors to act on the steering column. It is possible to add an actuator to make resistance and provide feedback to the driver and give realism to driving.

Some of the disadvantages of this technology are the security problems that involve completely eliminating the mechanical connection between the steering wheel and the steering column. The solution to this problem is to introduce redundant systems that ensure complete control at any time.

An example of this solution is the DAS (Direct Adaptive Steering) system from Infiniti Q50 [13], which, for safety regulations, has to be mechanically linked to the steering column parts. The system can act on the steering column through a clutch that is able to join the mechanical part with the steering rack instantly in case of failure in the electronic system. It is composed by three independent control units which work together and monitor each other, using the FlexRay-Bus.

### 2.2. Throttle-By-Wire Technology

The throttle is a mechanism used in internal combustion engines, due to it the air/fuel mixture flow is regulated by the throttle valve for petrol motors or the fuel volume, injected into the cylinders for diesel motors. In a conventional throttle, this regulation is made through a Bowden cable.

The electronic throttle [14] is a system which removes the mechanic connection between the throttle and the carburettor’s throttle valve. This is replaced by an electric connection through an electronic unit. When stepping on the throttle, a potentiometer is activated and a voltage is sent to the electronic unit, which batches the signal and sends other electric signals to the electronic throttle valve. Nowadays, the majority of fuel vehicles integrate this system. In the case of electric vehicles, the voltage generated for the throttle is directly sent to the power controller of the traction motor.

The main advantages of this technology are improved engine response, softener acceleration, better idling speed control, lower fuel consumption, better pollutant emissions control and easier cruise control integration.

### 2.3. Brake-By-Wire Technology

In 1918, Malcolm Lougheed created the hydraulic brake system [15]. Actually, it is the most used system in vehicle design. As vehicles gain weight and power, the driver should apply a bigger effort to brake. In 1927, Albert Dewandre developed the brake servo to help the driver in brake action; this system was sold that year by Bosch [16]. It is about a pneumatic system, which takes advantage of the vacuum that is produced in the petrol engine intake manifold when the drivers lift their foot from the throttle, to reduce the effort applied on the brake. In the diesel engine, the vacuum is created due to a pump joint to the engine.

The phasing out of traditional control systems replaced by electronic control systems has developed different Brake-By-Wire solutions. The electro-hydraulic system erases the brake servo’s need. It use sensors and actuator to measure the amount of pressure that the driver applies on the brake pedal and transfers this strength to the brake callipers through a master cylinder. By using control units and powerful actuators, the pressure applied among the brakes can be much bigger than traditional hydraulic brake’s systems.

This technology can be illustrated by iBooster [17], created by Bosch in 2013. This is a master cylinder where an electric motor which controls the brake amplification has been introduced. When emergency predictive brake system detects a dangerous situation, the iBooster might generate the higher pressure on an autonomous way in 120 ms. Subsequently, it is three times faster than traditional systems, achieving a braking distance reduction.

The future of braking systems will be the development of brake callipers where the whole actuation system is integrated. In this way, the hydraulic circuit is eliminated and an independent braking system is achieved for each wheel, electronically powered. This concept eliminates the hydraulic circuit, which has advantages in vehicle production, a better accident strength and improvement on the environment. Furthermore, as they are stand-alone units, if one of them fails, the others are available to stop the vehicle.

The vast majority of current vehicles integrate an EPB (Electronic Parking Brake) [18]. The rear callipers are electro-hydraulic and have a small electric motor attached to them, which presses the callipers to lock the parking brake when activated.

### 2.4. ROS Based Drive-By-Wire Technology

Nowadays, we can find Drive-By-Wire technology in new models developed by companies such as Tesla, Uber, Waymo, etc. and in some autonomous vehicle prototypes developed in universities and research centres. The automotive industry is really hermetic, and they have a great rejection to sharing and making public their breakthroughs. However, at present, there is an open source system for the development of robotic applications known as ROS (Robot Operating System), which is widely used by the scientific community and by some robotic companies, and that could be used in the automotive industry. However, there are currently very few ROS-based Drive-By-Wire projects, because large companies avoid losing control of their systems.

The AutonomouStuff company in the United States offers a ROS-based kit called PACMod, suitable with a Lexus RX and with several Polaris models. This kit offers the software and hardware necessary to drive these vehicles through ROS commands for acceleration, braking and steering wheel turn. The PACMod controller provides a communication interface between the ROS system and the vehicle’s CAN-Bus, making it possible to control the steering wheel, throttle, brake and gearbox. This company provides the PACMod for a price higher than $150,000, not including the cost of the vehicle [19].

Another example is the ADAS By-Wire kit, from the American company Dataspeed Inc., which was developed for a Lincoln MKZ in 2015 and currently can be installed in several vehicles. This kit also allows operating on the steering wheel, accelerator, gearbox and turn signals, through the vehicle’s CAN-Bus. The price of this kit, including installation, is $45,000 [20].

The UK StreetDrone company has developed a Drive-By-Wire system, integrated into the Nissan e-NV200 van, the Renault Twizy and the ZOE [21]. These platforms provide all the hardware technology necessary for autonomous navigation. Furthermore, the company offers the implementation of Autoware, at different levels according to user requirements. Autoware [22] provides all the necessary software for autonomous driving; it is based on ROS and is open source. It includes code for localisation, sensor fusion, object tracking, lane recognition and traffic lights, 3D maps, virtual reality, etc.

In addition, there are some other research projects on autonomous vehicles based on ROS. One of the most representative is the UK Autodrive project that started in 2018, where a large group of companies participate, such as Jaguar, Land Rover, Ford Motor Company, Tata Motors European Technical Center and so on [23].

This work has been developed within the context of the Tech4AgeCar project, in which a cheap and open source self-driving electric vehicle should be designed. Given the shortage of existing open source proposals in the state of the art and their high prices, we decided to automate an open source electric vehicle chassis from scratch. The main aim was to develop a hardware platform that allowed verification and validation tests of our autonomous navigation proposals in real environments.

In this paper, we present the development of an efficient and robust Drive-By-Wire system, implemented for our electric vehicle and suitable for the navigation architectures developed within the project. Our system will be controlled by a stand-alone unit based on ROS, which will allow controlling the vehicle through linear and angular speed commands that will be sent through a local network. This approach will provide an open source hardware platform, which will allow its reproducibility by third parties.

## 3. Techs4AgeCar Project

The main aim of the Techs4AgeCar project is to develop an open source autonomous electric vehicle. As commented in the previous sections, this approach has advantages, because it allows the contribution of freelance developers, keeping a constant update of both the software and the hardware, reducing in this way the project’s costs exponentially. This section reviews the vehicle characteristics and the different modules of our navigation architecture in order to put in context our Drive-by-Wire system development.

### 3.1. The Vehicle

Due to the high costs of commercial solutions for having an automated vehicle and the fact that these solutions consisted of closed systems, we decided to automate an open source chassis. Currently, the two most important open source platforms for electric vehicles are POM, developed by Renault and based on Twizy [24], and TABBY EVO, developed by the company Open Motors [25].

After analysing their characteristics, the TABBY EVO chassis was chosen for this project since it offers higher performance. It is an open source platform whose plans and electric diagrams are available to be downloaded and being freely modified. This allows developers to incorporate their technological innovations and to create their own brands, accelerating the expansion of electric vehicles.

**Figure 1:**
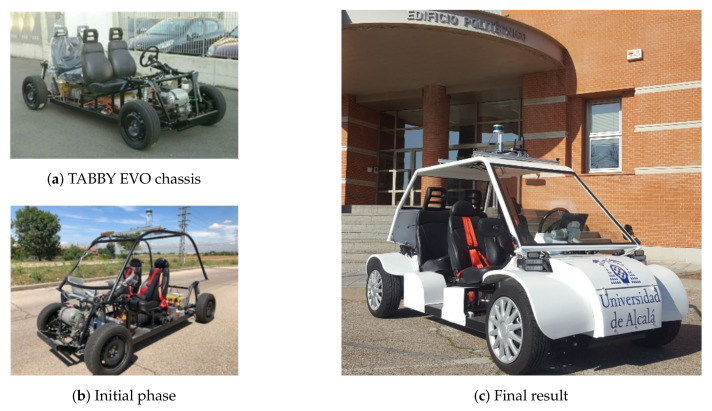
Techs4AgeCar’s vehicle progress.

The chassis was initially equipped with 4 seats, a 19-kW asynchronous AC motor, an AC-L1 controller from the Italian group SME, a BMS SCC24 from the XBW brand and a ZIVAN NG3 charger that sends 80 V and 25 A to the batteries (Figure 1a). The technical characteristics of the vehicle are presented in Table 1.

In the first phase of the project, the battery pack was installed in the vehicle and a tubular steel chassis was designed and implemented for the upper part in order to place the sensors (Figure 1b).

Subsequently, a steel bodywork was designed and manufactured on this chassis, the mechanical steering column was replaced by an electric one and the necessary hardware for autonomous navigation was added, which is explained in the next section.

An aluminium roof rack was built and installed on the top of this structure, where all the sensors, both perception and localisation, were housed. The final result of the vehicle is shown in Figure 1c. The roof rack has an anchoring system that allows it to be easily removed and put on; in this way, it is possible to store all the sensors in a safe place after finishing using the vehicle.

**Figure 2:**
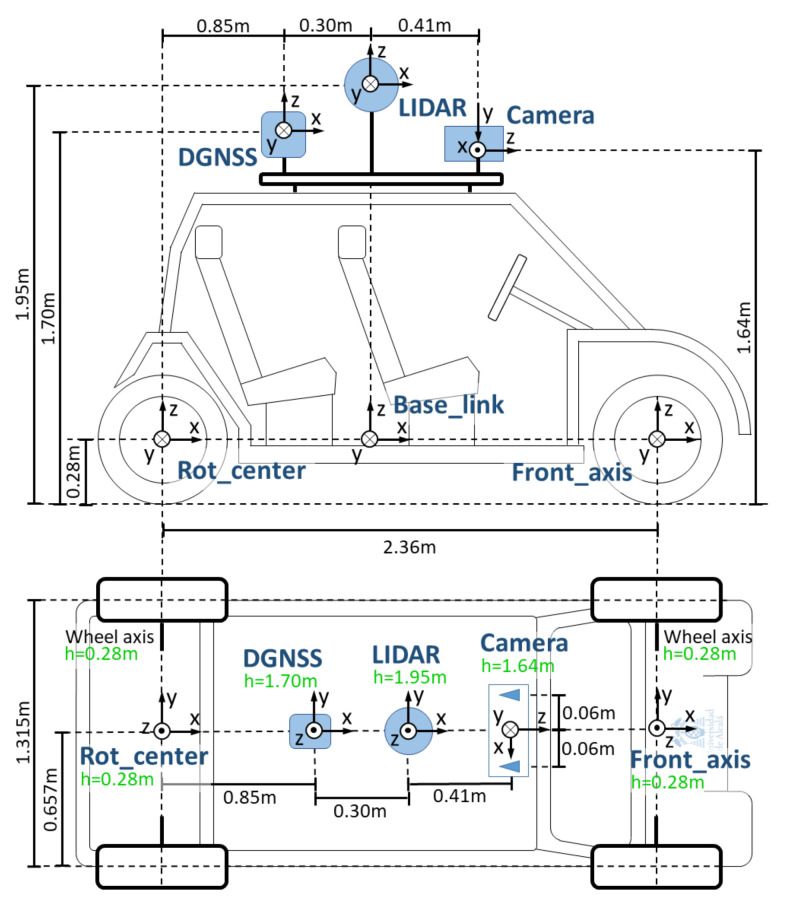
The arrangement of the Techs4AgeCar sensory system.

Figure 2 shows the arrangement of the sensory system in the vehicle, with the coordinate axes of each sensor. As can be seen, it is based on a stereo camera, a LIDAR and a DGNSS.

### 3.2. Autonomous Navigation Architecture

The Techs4AgeCar autonomous navigation is based on a modular architecture, in which each module processes the information independently and asynchronously. The modules are implemented in nodes and the communication among them is asynchronous and based on topics, through the IPC (Inter-Process Communication) of ROS [3].

**Figure 3:**
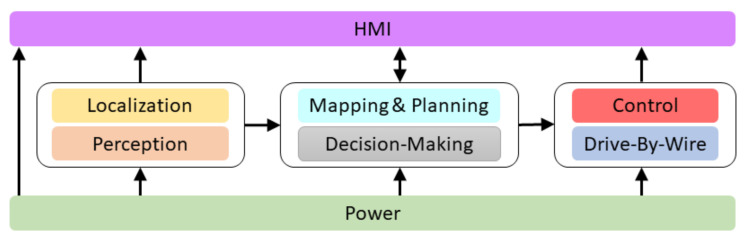
Techs4AgeCar autonomous navigation architecture.

Figure 3 shows the navigation architecture that is divided into eight modules: Power, Localisation, Perception, Mapping and Planning, Decision-Making, Control, Drive-By-Wire and HMI (Human–Machine Interface). Each of them is explained in detail below.

**Figure 4:**
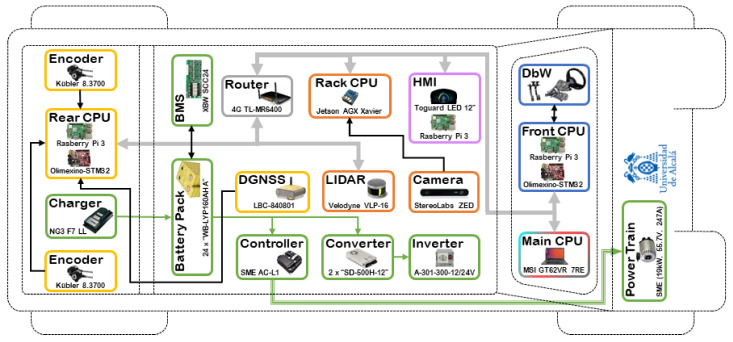
Techs4AgeCar hardware architecture.

Due to the high processing requirements and response speed of the autonomous system, the processes are divided into different CPUs (Central Processing Units). Actually, there are five main CPUs in the vehicle, as shown in Figure 4. A colour code is used to classify each hardware device among the eight modules of the navigation architecture.

Some CPUs are based on the Raspberry Pi 3 model B, due to the need to choose an embedded system able to support ROS, with low consumption and low cost. It is a low-cost SBC (Single Board Computer) developed by the Raspberry Pi Foundation. It is s a tiny 85 mm × 54 mm motherboard that houses a Broadcom BCM2837 chip with 1.2 GHz ARM Quad Core processor, VideoCore IV GPU and 1 GB of RAM [26]. In the vehicle, there are three Raspberry Pi computers: one in the rear responsible for processing the Localisation Module, one in the front that controls the Drive-By-Wire Module and one in charge of processing the HMI Module.

The CPU responsible for processing the images captured from the camera is a NVIDIA Jetson AGX Xavier, and it is placed on the roof rack.

Finally, the main processing unit is a laptop. It is in charge of the mapping, planning, 3D perception and decision-making processes. This is based on a MSI GT62VR 6RE Dominator Pro model, an upmarket gaming laptop with a GTX 1070 graphics card.

### 3.3. Power Module

The Power Module is formed by a three-phase asynchronous traction motor, the controller, the batteries, the BMS and the charger.

The traction motor is the model MT906B [25] of the Italian group SME. It is a three-phase asynchronous motor, with a nominal values whose voltage is equal to 80 Vac and current is equal to 235 A, providing a nominal power of 19 kW. The maximum power offered by this motor is 29.5 kW at a speed of 2500 rpm and a maximum torque of 128 N·m at 500 rpm. The maximum motor efficiency is 91.97% at 5000 rpm.

The controller is the device responsible of extricate energy from the batteries and supply it to the motor, according to the conditions and instructions indicated by the driver. The controller model is the SME AC-L1, from the same motor’s manufacturer. The maximum current that this motor can absorb is 400 A, electronically limited by the controller.

The battery sends 82 V and is composed of 24 cells of LiFePO4 3.4 V 160 Ah. The lithium iron phosphate battery (LiFePO4) is a lithium ion battery with a lithium iron phosphate cathode. Compared to lithium cobalt oxide batteries, they are more stable, safer, more durable and do not present a risk of combustion. Therefore, they are suitable for automotive applications.

The BMS (Battery Management System) is in charge of managing and monitoring the charge and discharge of the battery, depending on parameters such as the maximum charge or discharge current, operating temperature, maximum charge voltage and minimum discharge voltage of the cells. In addition, it communicates with other control units outside the battery, such as the traction motor driver that controls the vehicle speed, energy recovery in braking and so on.

Additionally, there are the converters and the inverter, which provide the electrical power to all the navigation modules.

### 3.4. Localisation Module

This module is in charge of locating the vehicle on a map with centimetric accuracy in real time. A precise and robust localisation module is vital for our autonomous navigation system.

The vehicle’s pose estimation is based on the merging of the positions provided by a DGNSS (Differential Global Navigation Satellite System) receiver and by an encoder-based odometer.

GNSS (Global Navigation Satellite System) technology includes GPS, Glonass and Galileo systems. Errors of these systems imply low accuracy in the real time pose estimation. To improve the accuracy and reliability of the data, DGNSS and RTK (Real Time Kinematic) techniques are used [27].

DGNSS and RTK use two GNSS devices working at the same time. One of them is located in a known position and the other one is on board the vehicle. Consequently, there is a motionless base station, at a reference point with known coordinates, and a mobile unit, in motion, at unknown coordinate points. With these technologies, it is possible to estimate the positioning errors and fix them in real time, obtaining centimetric accuracy.

**Figure 5:**
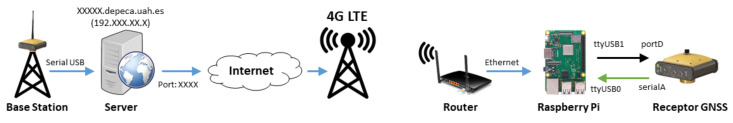
RTK Localisation structure.

In this project, we use RTK technology, which achieves greater accuracy due to the measurement of the satellite signal’s carrier phase, as the basis for calculating distances and the use of two receivers. The receivers used are the Topcon Hiper Pro model [28]. The two 40-channel GNSS RTK devices can track GPS and Glonass signals, with integrated UHF radio. The hardware structure of the Localisation Module is shown in Figure 5.

The stationary receiver, or reference base, is located on the rooftop of the Polytechnic School of the University of Alcalá, whose position is known exactly. This receiver analyses the signals from all visible satellites and compares the position calculated from this information with the prior known position. This deviation is caused by errors associated with the environment, and these errors use to be common in a region close to the receiver.

**Figure 6:**
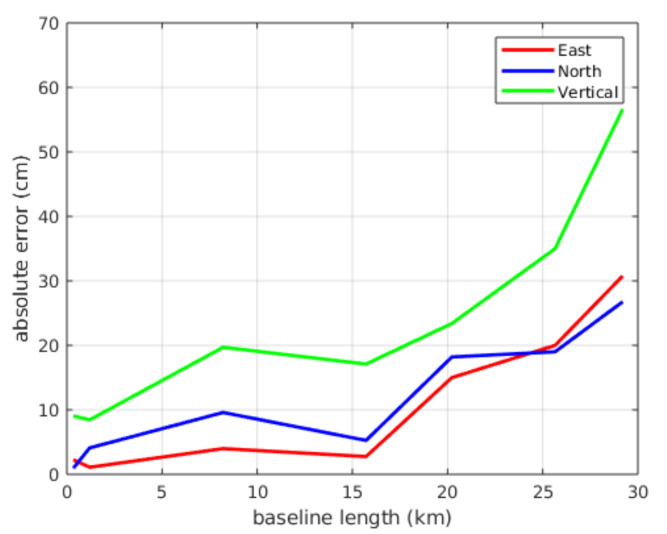
RTK positioning accuracy.

The RTK system was tested and the positioning errors shown in Figure 6 were obtained. It can be seen that positioning accuracy decreases when increasing distance to the base; thus, it was decided to install our own RTK base within the campus where the tests are carried out.

**Figure 7:**
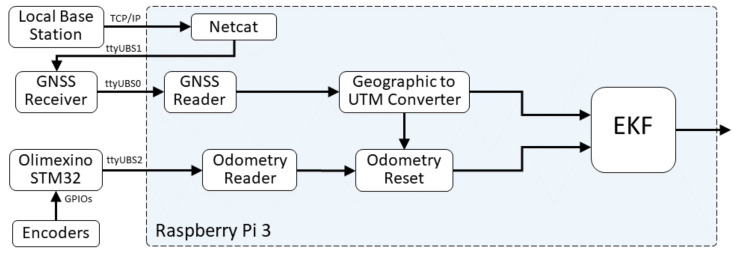
Localisation Module’s diagram.

The base station’s DGNSS receiver generates the differential corrections for the mobile device at a frequency of 10 Hz. These data are read by a server and sent through a TCP/IP connection to the vehicle, which has a 4G router with Internet access. On the other hand, in the vehicle, there is a Raspberry Pi 3, in charge of processing the Localisation Module. This board connects with the Netcat tool to the server and sends the data received through a USB serial port to the vehicle’s DGNSS receiver, which obtains the corrections, fixes its observations and sends the corrected positions to the Raspberry Pi 3 through another USB serial port, as depicted in Figure 7.

Communication with the DGNSS receiver is done using NMEA (National Marine Electronics Association) commands [29]. This device sends the positions using the GPGGA (Global Positioning System Fix Data) statement [30], which contains the latitude and longitude, according to the following structure:

$GPGGA, 134658.00, 5106.9792, N, 11402.3003, W, 2, 09, 1.0, 1048.47, M, −16.27, M, 08, AAAA*60

DGNSS receivers provide their measurements in geographic coordinates, where any point on the surface is characterised by two angular values: latitude and longitude, expressed in degrees, minutes and seconds. However, due to the complexity in distances’ calculation of angular magnitudes, it is common to work with UTM (Universal Transverse Mercator) coordinates. As result, working with distances is greatly simplified by using a Cartesian system.

Since the Earth is not a perfect sphere, there are different techniques for converting geographic coordinates to UTM and vice versa. In this project, this conversion is done using the libraries implemented in the ROS geodesy package [31].

On the other hand, odometry is used to estimate the position of the vehicle by measuring its rear wheels motion. It is based on the integration of the rotation speed of the wheels over time, which implies an unavoidable accumulation of errors. This solution is cheap, simple to implement and very accurate in short periods of time where the DGNSS signal quality is unsatisfactory.

Two Kübler model 8.3700.1322.0360 encoders were installed on the rear wheels. It is an optical incremental encoder of 360 pulses per turn, built with a 37-mm diameter carbon fibre reinforced plastic housing that makes it extremely robust and resistant [32].

Firstly, the pulses of the two encoders are read by an Olimexino STM-32 development board. The use of this board is important, since it has a RTC (Real Time Clock) module that allows capturing the encoder information without losing pulses. The Olimexino STM-32 sends the pulses read from the encoders in a determined time interval, using a serial communication protocol.

**Figure 8:**
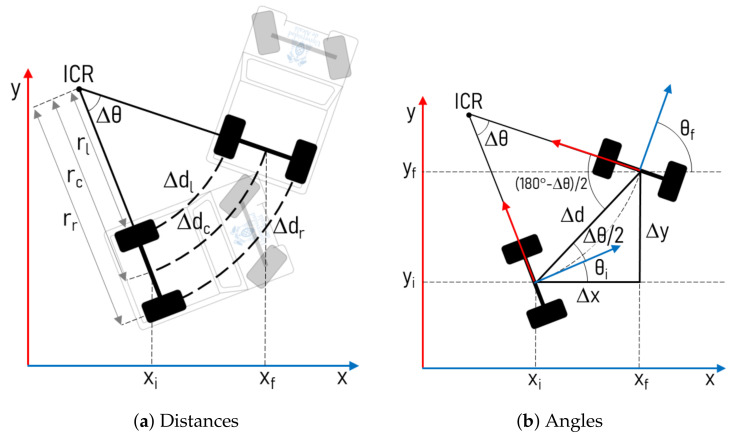
Odometry Model.

Secondly, a Raspberry Pi 3 receives the information from the encoders, through another USB Serial port, and performs the odometry to obtain the vehicle’s 2D pose. These calculations are referenced to the centre point of the rear axis, supposing that the movement is performed in a plane and it follows the classical odometry model for differential traction vehicles, as shown in Figure 8.

The Raspberry Pi 3 performs localisation estimations periodically, with a sampling time of 100 ms. At the beginning of each iteration, data are captured from the DGNSS receiver and the encoders. Subsequently, the vehicle pose is obtained from the UTM coordinates; at the same time, the pose is calculated according to the odometry model, the encoders’ information and the position obtained in the previous interaction. If the DGNSS signal quality is optimum and the RTK system obtains an accurate pose, the odometry position is reset to the DGNSS pose.

The poses obtained from the DGNSS and the odometry are merged using the robot_localization package provided by ROS. This package is responsible for capturing sensor data and estimating the vehicle’s pose using an EKF (Extended Kalman Filter) [33].

The Kalman Filter is a recursive estimation algorithm that is capable of predicting the state of a variable from an initial estimate, the measurements provided by the sensors and the uncertainty in the measurement of these sensors. Because it is recursive, it does not need to store the information from all the previous steps. On the contrary, the estimation at each moment is the result of the estimation at the previous moment, which gives it great power and facility of programming. In addition, one of its greatest virtues is that it is capable of considering, at each moment, whether the prediction is more reliable than the measurements and vice versa, and which of the sensors is more reliable. For more details on RKF, see [27].

**Figure 9:**
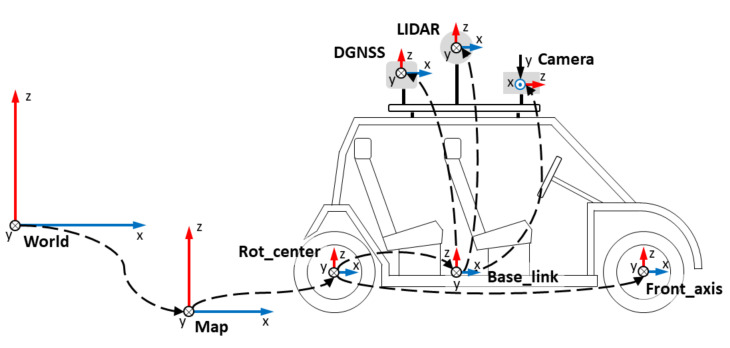
Tech4AgeCar’s frames.

In ROS, the management of the different reference systems, or frames, is crucial. When locating the vehicle, it is important to use the correct reference system, taking into account the base of the vehicle and the different sensors found in it. Each of these elements consists of a different coordinate system, and it is necessary that all the frames of the same system belong to the same transform tree. These trees specify the relationship between the frames of different elements, allowing ROS to be able to calculate the transforms between them. Figure 9 shows the frames currently used in this project.

### 3.5. Perception Module

The Perception Module is necessary for driving in a safety way. For that purpose, this module analyses the environment that surrounds the vehicle, detecting and recognising the objects that are in it, both static (roads, trees, traffic signs and so on) and dynamic (other cars, pedestrians, cyclists and so on). The perception of the environment is based on the fusion of the point cloud obtained with a LIDAR and the RGB images taken by the camera.

**Figure 10:**
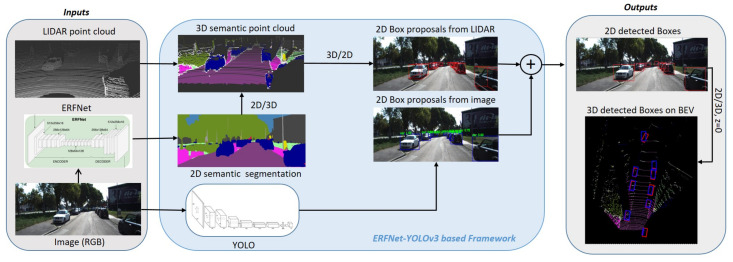
Perception Module structure.

The images are processed using two CNN (Convolutional Neural Network): YOLOv3 [34] to detect objects in RGB images and ERFNet (Efficient Residual Factorised ConvNet) [35], developed in the project, to obtain a semantic segmentation of the image in real time. The diagram of the Perception Module is shown in Figure 10.

Detection of 3D objects in BEV (Birds Eye View) from RGB and LiDAR images is performed as follows. The captured image is processed by a YOLOv3 quickly and accurately. The outputs of this detector are the bounding boxes with the objects detected in the image. Meanwhile, the ERFNet obtains a semantic segmentation of the same RGB image. The segmented pixels are merged with the LIDAR 3D point cloud, obtaining a coloured 3D point cloud, in the camera’s field of view, where each colour represents a class of obstacle (vehicles, pedestrians, trees, etc.). Each colour cluster is modelled by a 3D bounding box. The point cloud outside this field of view remains unsegmented.

Finally, a fusion of the objects detected by vision and by the LIDAR is made. Furthermore, the 3D position, volume and class of each detected object are obtained. A tracking based on a Kalman Filter [36] is performed for each of them, with the purpose of obtaining the prediction of their position.

For a more detailed explanation of the method, we refer the reader to the following reference of the authors [37].

### 3.6. Mapping and Planning Module

**Figure 11:**
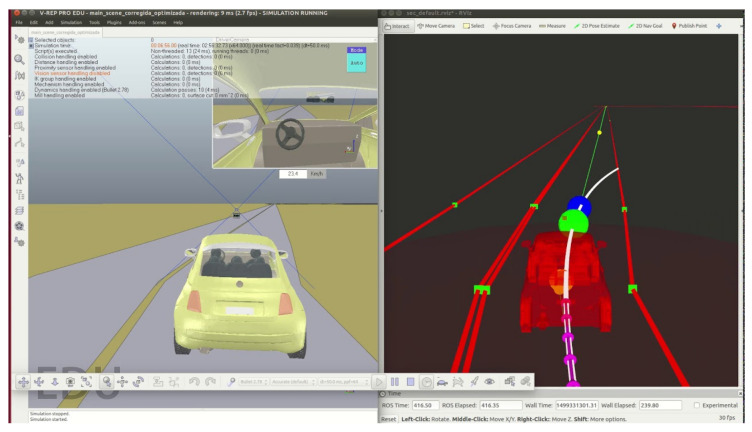
Mapping and Planning Module.

Our navigation architecture is based on a priori map. The maps implementation has been carried out applying the lanelet approach [38]. Using the open source JOSM tool, a map of the University of Alcalá Campus has been obtained to validate our navigation proposals. The lanes and the connections between them have been manually delimited, including information from traffic regulatory elements (such us stop signs, yield signs, intersections, roundabouts, etc.), to generate an enhanced map useful for navigation, as shown in Figure 11.

After receiving an origin and arrival point, a lanelet route is obtained using an A* algorithm from the maps. From this sequence of lanelets, the Global Planner generates a path, made up of multiple waypoints, that the vehicle must follow.

These waypoints are defined by a position on the map and they are created in the middle of the lanes. The distance between them is selected based on the length and the radius of curvature of the lanelets.

A data structure is created and includes the shortest route between any two points on the map, and it sends it to the local path planner. In addition to the trajectory, contiguous lanes are provided for overtaking, intersection lanes for crossover manoeuvre and the position of the regulatory elements.

For a more detailed explanation of the method, the reader is referred to [39].

### 3.7. Decision-Making Module

The decision of the manoeuvre to be carried out each moment depends on the Planning and Perception Modules, which allows analysing the vehicle surroundings and making the appropriate decision to follow the global planning safely complying with traffic regulations. In this module, different driving behaviours are implemented, which define the trajectories to be follow and their temporisation (lane keeping, stop, yield, pedestrian crossing and so on).

The sequence of actions and events, that define a behaviour, is implemented through Petri nets using the RoboGraph tool [40]. The definition of Petri nets is done using the RoboGraph Editor tool while its execution is carried out with RoboGraph Dispatch. The interaction with the modules of the architecture is done through the publication and subscription of messages in ROS. In this way, problems that may occur in a module, such as a lockout, will not cause the Dispatch’s lockout.

The tasks associated with a behaviour are defined using an editor of interpreted Petri nets and saved in an XML file. Dispatch is the execution program that is in charge of loading these files and executing the Petri nets, each time that a node requests it, by sending the corresponding message. On the other hand, there is a program to monitor the evolution of the Petri nets in progress, very useful for debugging and tracking.

For a more detailed explanation of the method, we refer the readers to our publication [2].

### 3.8. Control Module

The Control Module or Local Navigation generates local paths to reach the intermediate waypoints calculated by the planner, avoiding obstacles, and calculating the linear speed and curvature necessary to follow the generated local path.

The Control must guarantee a safe navigation regardless of the choice made by the Decision Module. When an obstacle is detected, the vehicle has to stop or to avoid it in real time. Local Navigation is based on two simple and fast algorithms, which have been efficiently implemented in computers with low processing capacity.

The Pure Pursuit algorithm [41] has been used to track the trajectories. This algorithm searches for the closest waypoint in the direction of the route and tries to reach it. It calculates the curvature that drive the vehicle to the chosen point on the route and sends it to the reactive or obstacle avoidance module.

The method used to avoid obstacles is an adaptation of the BCM (Beam Curvature Method) [42]. The outputs of this module are the linear speed and curvature that are sent to the Drive-By-Wire Module. For a more detailed explanation of the method, the reader is referred to [37].

### 3.9. Drive-By-Wire Module

The Drive-By-Wire Module receives the setpoints sent by the Control and transforms them into steering wheel turn and drive wheels turn speed, following the Ackermann model. Consecutively, it generates the necessary control signals to reach these setpoints, and it sends them to the actuators that generate the vehicle’s movement. This module is explained in detail in the next section.

### 3.10. HMI Module

**Figure 12:**
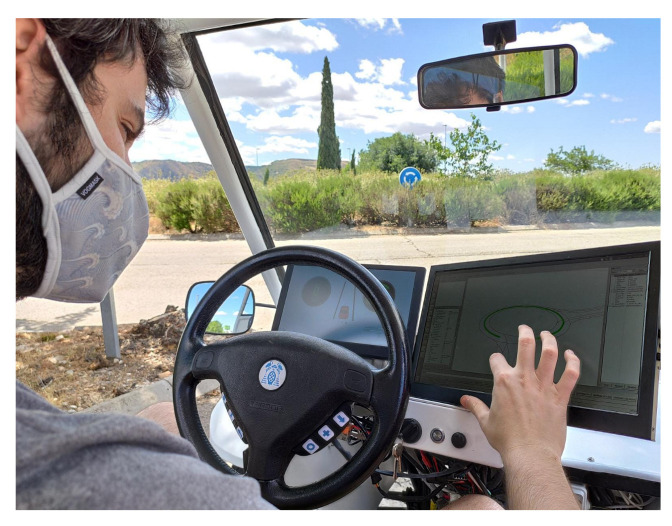
HMI.

The HMI (Human–Machine Interface) allows the driver to interact with the vehicle, select the destination and view the route and vehicle’s status on a screen, as depicted in Figure 12.

The HMI implemented on the car has two screens. The first one (on the left) allows the driver to know the velocity, battery state, localisation information (as number of satellites or the state of DGNSS receiver), lights state and information provided by the Perception Module about next objects on the path. The second one (on the right) is a touch-screen which is used to launch the path.

## 4. Drive-By-Wire System

**Figure 13:**
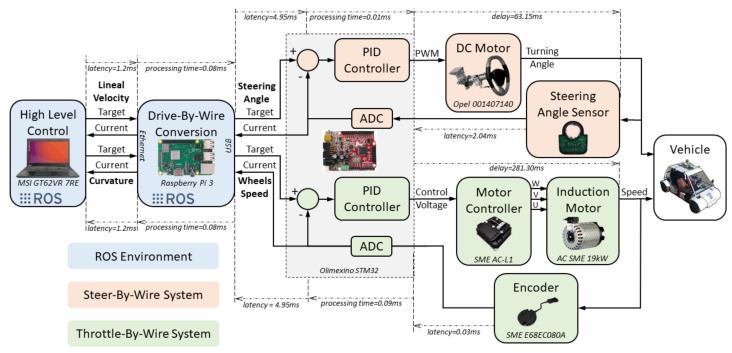
Latency of the Drive-By-Wire system.

The architecture of our Drive-By-Wire system is shown in Figure 13, and it works in the following way. Firstly, every module that compose the Drive-by-Wire system has to be connected in the same local network with the other modules of our vehicle’s navigation system. Through this network, two setpoints are sent, the linear speed and the curvature, which are read by the Drive-By-Wire module and converted into steering wheel turning angle and drive wheel turn speed.

These control values are sent to a development board where two PID controllers have been programmed, one for each controlled variable. The steering wheel turning angle is introduced into the Steer-By-Wire system as a reference signal. To close the control loop, the steering wheel angular position is obtained through a magnetic sensor and the wheel is moved with a motor until the desired position is reached.

The required wheel turn speed is achieved through the vehicle’s traction motor controller until the set speed is achieved due to an encoder that measures the motor’s actual speed continually.

This Drive-By-Wire system was designed in six phases. The first one was the Steer-By-Wire module. In this phase, firstly an absolute angular position sensor was developed. Subsequently, the mechanical steering column of the TABBY EVO was replaced by an electric one, built for an Opel Corsa power steering motor where the position sensor was attached. This whole system was controlled from a DC motor driver and a development board.

The second phase consisted on the development of the Throttle-By-Wire module, since it was an electric vehicle, this phase was easy to achieve. The vehicle’s throttle is controlled by a direct voltage that is sent to the asynchronous AC motor controller. This control voltage is generated from a DAC (Digital Analogue Converter), rather than the throttle pedal. An encoder was installed in the traction motor to feed back the control loop.

At this point in the project, we realised that the braking achieved with the regenerative brake of the electric motor was enough for the autonomous navigation tests carried out with our vehicle. Therefore, we decided that the Brake-By-Wire module was not necessary at the moment, although we intend to develop it in the near future.

In the third phase, we designed a switching module to activate autonomous driving and to take manual control of the vehicle at any time the driver to require it and in a safety way.

In the fourth and fifth phases, all the modules of the system were grouped within a single development board. To achieve real time execution of all the processes, the FreeRTOS operating system was used.

Finally, in the sixth phase, the ROS-based module was implemented, which allows the Drive-By-Wire system to be controlled and communicated with the vehicle’s navigation system.

### 4.1. Steer-By-Wire System

The Steer-By-Wire module is composed by three modules: the position sensor, the electric steering column and the DC motor driver.

**Figure 14:**
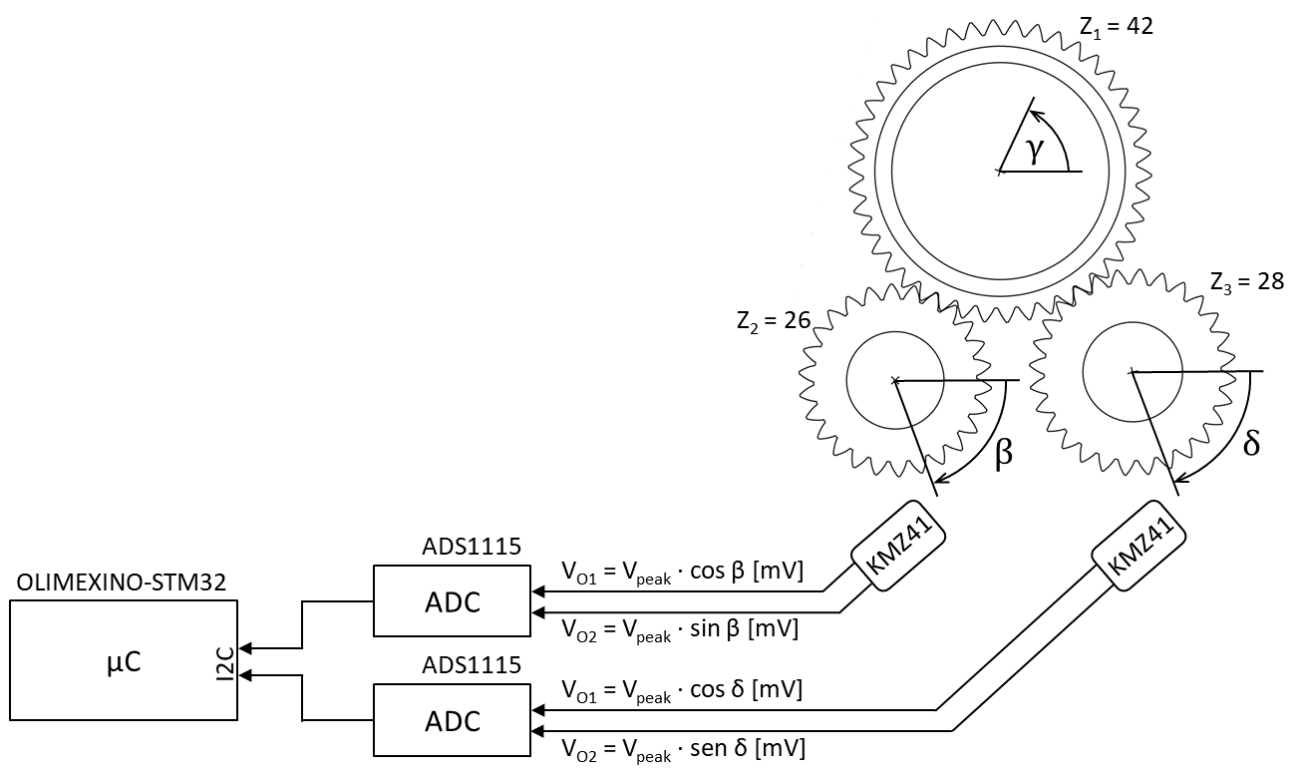
Angular position sensor diagram.

Although there are different commercial position sensors in the market, we decided to design our own sensor to have full control over it. The position sensor is composed by a magnetic absolute angular position sensor, which provides the angle of the largest gearwheel, from the secondary angles of two small gearwheels attached to the main one, as shown in Figure 14.

The angle provided by the sensor (γ) is calculated based on the subtraction of the two secondary angles (β and δ), from Equations (1) and (2), where Ω is the periodicity of the magnetic sensors that measure the secondary angles:

(1) $$\gamma =f\left(\beta -\delta \right)\cdot k=\frac{f\left(\beta -\delta \right)\cdot \gamma_{max}}{\Omega }$$

(2) $$f\left(\beta -\delta \right)=\left\{\begin{array}{ccc} \left(\beta -\delta \right) & if\; & (\beta -\delta )\geq 0\\ \left(\beta -\delta +\Omega \right) & if\; & (\beta -\delta )<0 \end{array}\right.$$

Finally, the maximum angle ($\gamma_{max}$) that the sensor will provide is calculated from the least common multiple (LCM) of the number of teeth of the secondary gearwheel ($Z_{2},\;Z_{3}$), the sensors’ periodicity (Ω) and the number of teeth of the main wheel ($Z_{1}$), according to the following equation:

(3) $$\gamma_{max}=\frac{LCM(Z_{2},Z_{3})\cdot \Omega }{Z_{1}}$$

To measure the angles of the secondary wheels, two NiFe-based thin-layer magnetoresistive sensors (also called Permalloy) or AMR (Anisotrop Magneto Resistive) are used. The NXP Semiconductors KMZ41 [43] sensor provides two analogue output signals of sine and cosine of the angular position of the surrounding magnetic field from 0° to 180°.

The output voltages provided by the KMZ41 sensor range ±80 mV. These voltages are too small to be sampled by the ADC of a development board. Therefore, the Texas Instruments ADS1115 [44] external digital analogue converter is used, which provides four 16-bit ADCs, allowing a resolution of up to 7.81 μV. Additionally, it is connected by I2C and has two measurement modes, single ended and differential. The use of I2C and differential mode provides great immunity to noise despite working with such small voltages.

**Figure 15:**
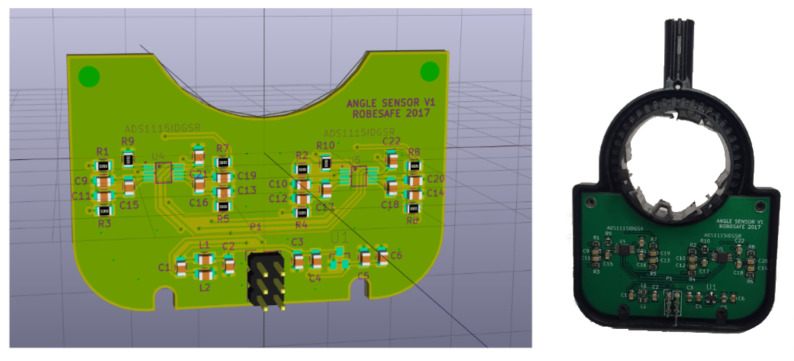
Angular position sensor’s PCB design.

The PCB (Printed Circuit Board) corresponding to the angular position sensor’s diagram was designed and the gerbers (file format for the manufacture of printed circuits) were sent to a PCB manufacturing company. The necessary components were purchased, the manual assembly of all the components was carried out and the developed boards were validated. The final result can be seen in Figure 15.

**Figure 16:**
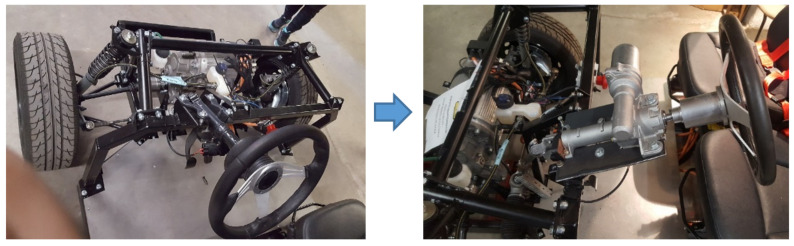
The replacement with electric steering column.

As shown in Figure 16, the mechanical steering column of the TABBY EVO platform was replaced by an electric one, in order that the turn of the steering column could be activated by the driver or by a motor.

In addition, between the steering wheel and the motor input we can find a torque sensor, constructed by strain gauges whose resistance fluctuates with the applied torque. To obtain a measurement of voltage in accordance with torque, it is necessary to connect the sensor to an electrical circuit capable of measuring variations in the resistance of the gauges from a supply voltage. This sensor uses four strain gauges electrically connected between them in what is known as the Wheatstone bridge circuit.

The driver used is the IBT-2 [45] model based on the Infineon BTS7960 chip to control high-power direct current motors, capable of supplying up to 43A of current at a supply voltage of 6–27 V.

The driver’s logic level inputs work with voltages from 3.3 to 5 V, making it suitable for most microprocessors. In addition, it allows controlling the motor speed through PWM (Pulse Width Modulation) with a maximum frequency of 25 kHz.

### 4.2. Throttle-By-Wire System

This system consists of three modules: the AC traction motor, an encoder attached to the motor and the AC motor controller. The traction motor is the model MT906B [25] of the Italian group SME. The controller for this motor is model AC-L1 from the same manufacturer. The traction motor has an integrated quadrature encoder, the model E68EC080A from SME, which provides a resolution of 64 pulses per revolution. To read the encoder signals, a conditioning stage must be added to convert these signals into simple logic levels. In this case, NAND logic gates with Schmitt trigger are used, which apply an electronic hysteresis.

To perform this function, the Texas Instruments CD4093B [46] is used, a CMOS family integrated circuit consisting of four two-input NAND gates with Schmitt trigger action on both inputs. The signal of each encoder channel is taken to the two inputs of a NAND gate. Therefore, at the output, the signal is inverted, which in this case is not important since only the frequency of the pulses will be measured.

The throttle voltage that is sent to the controller has a range from 0.5 to 4.5 V. The Olimexino-STM32 DAC has a reference voltage of 3.3 V, therefore, an external DAC is required. The MCP4725 manufactured [47] by Microchip, which has a 12-bit resolution, was used. It is controlled thanks to the I2C bus, therefore, reading it is an easy process. The MCP4725 supply voltage is from 2.7 to 5.5 V. The maximum voltage it can provide is Vcc. Powered at 5 V, its 4096 levels (12 bits) provide an accuracy of approximately 1 mV.

### 4.3. Manual/Automatic Switch System

This module allows the driving mode to be switched between manual and automatic, acting as a multiplexer between the signals generated by the vehicle’s actuators and the signals generated from the automatic control via software.

This system, in addition to being essential to operate in both driving modes, is necessary for security reasons. Current legislation requires the driver to watch out and be prepared take control of the vehicle at any time. For this reason, the designed system gives the vehicle’s controls to the driver at the time he requires it.

To switch the driving mode, pressure must be applied to the throttle pedal, brake pedal or steering wheel. In this way, whenever the driver detects an unusual situation in which the vehicle is navigating automatically, but incorrectly, the driver can take the control.

In addition, for security, the system is not implemented in a microcontroller, which is more susceptible to possible failures such as restarts, system delays, etc. Therefore, it is based on logic circuits; in this way, the system starts at the instant the vehicle is powered without the need to wait for a setup time.

**Figure 17:**
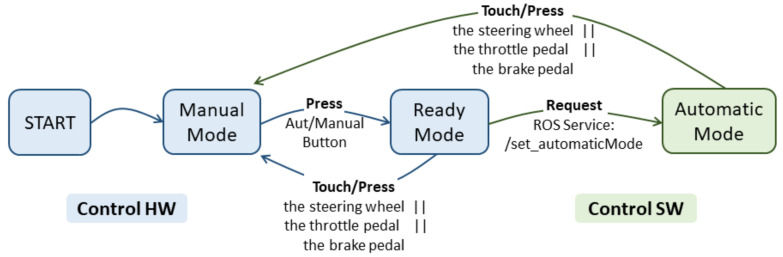
Working of the Manual/Automatic Switch Module.

Figure 17 shows the working flow of this system. Vehicle starts in Manual mode. Pushing the Auto/Manual button on the control panel will enter Ready mode. In this mode, the vehicle is already prepared for autonomous driving in terms of hardware elements.

For software security, a request for ROS service must be sent to switch to Automatic mode, which is explained below. As soon as the brake pedal, the throttle pedal or the steering wheel is actuated, the vehicle changes into Manual mode. This system is based on a sequential logic circuit.

### 4.4. Drive-By-Wire Hardware

The whole system, explained up till now, is controlled with an open source Olimexino-STM32 [48] development board. It has the same physical design as a conventional Arduino board, but it is more powerful and is prepared to be programmed and used in a very similar way, using the Arduino IDE.

**Figure 18:**
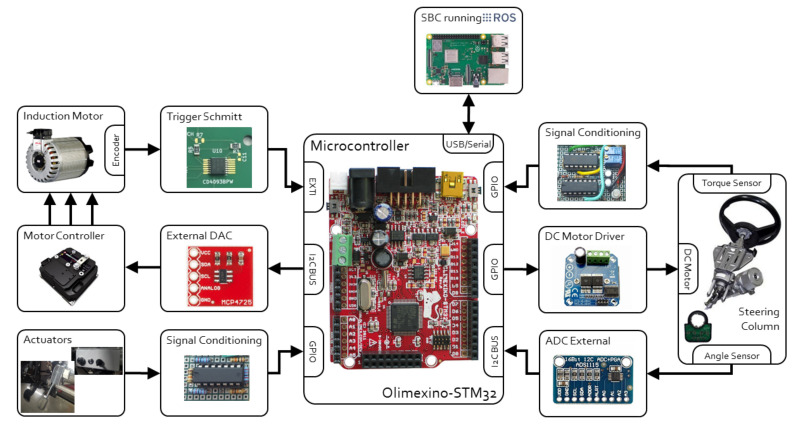
Drive-By-Wire’s hardware structure.

Figure 18 shows the diagram of the Drive-By-Wire hardware structure, with all the devices explained above as well as their connections.

To implement this hardware, in the first phase, DIP (Dual In-line Package) components placed on shields or modular circuit boards mounted on top of each other were used to give extra functionality to the development board. After carrying out the necessary tests, an initial scheme of the Drive-By-Wire system was obtained, from which a PCB (Printed Circuit Board) was designed, based on an Arduino shield and SMD (Surface Mounting Device) components.

**Figure 19:**
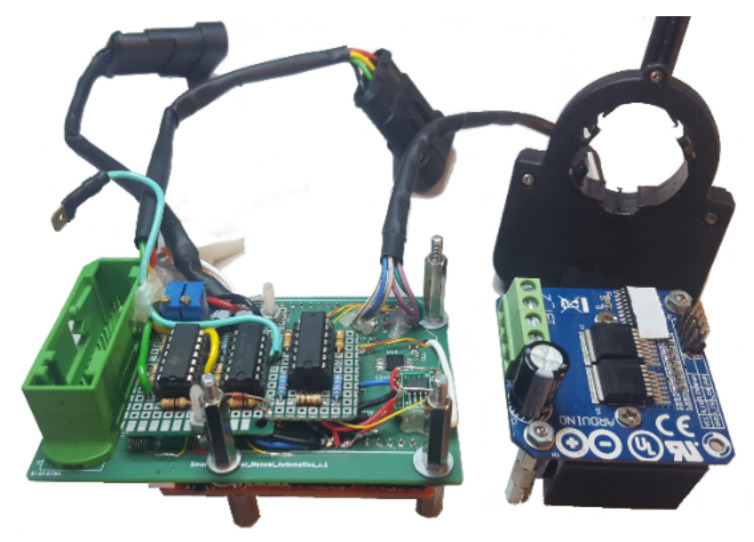
Current state of the Drive-By-Wire hardware.

Following the PCB design, the gerbers were shipped to a PCB manufacturing company and all SMD components were manually assembled. From this initial board, modifications and new components were implemented until obtaining the current system, as shown in Figure 19.

### 4.5. Drive-By-Wire Software

To program the Olimexino-STM32, the Arduino IDE (Integrated Development Environment) [49] was used. For this, a repository is available with the STM32 compatibility code with Arduino that is open source and based on the work developed by the company Leaflabs for the Maple board [50].

Using the Arduino IDE to program the STM32 is a great advantage from the point of view of comfort and utility, because it allows using a simple and well-known environment. In addition, it allows developers to take advantage of many tools created by the Arduino community. One of these tools is the FreeRTOS library that can be added to the Arduino IDE [51].

FreeRTOS is a multitasking RTOS (Real Time Operating System) to manage hardware resources and the execution times of the different tasks implemented for an application running in microcontrollers or small microprocessors.

**Figure 20:**
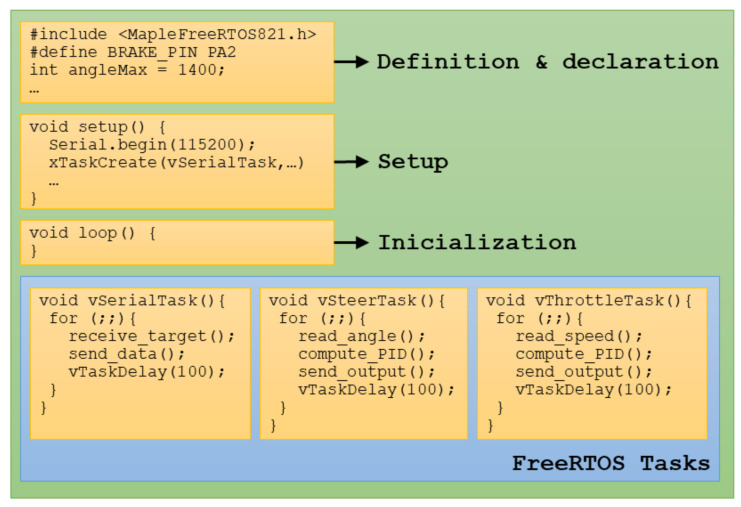
Arduino code structure for the Drive-By-Wire system.

Figure 20 shows the structure of the Arduino code compiled and loaded on the Olimexino-STM32 board. Firstly, the necessary libraries must be installed and declared. Next, all the necessary variables are defined and the elements are configured and initialised in the setup () function. The loop () is the main function in an Arduino sketch but in this case the functions will be executed within the three FreeRTOS tasks created: Communication, Steer-By-Wire and Throttle-By-Wire.

**Figure 21:**
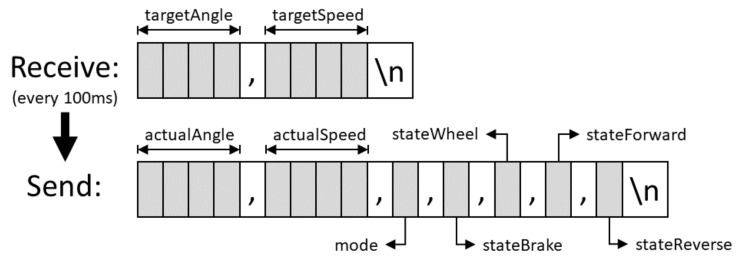
Serial communication protocol for the Drive-By-Wire system.

The development board communicates through USB (Universal Serial Bus) with other devices by using the communication protocol is shown in Figure 21.

Every 100 ms, the communication task is run, which checks whether new setpoints have been received, verifies that they are in the correct format and if they are within defined ranges. Then, vehicle’s current state is sent through the same port: angular position of the steering wheel and turn speed of the drive wheels.

The states of five signals are also sent, which define the state of the vehicle and are read by a GPIO port of the microcontroller. If a setpoint is not received during one second, for safety reasons, the vehicle will stop and the steering wheel will be brought to position 0°.

**Figure 22:**
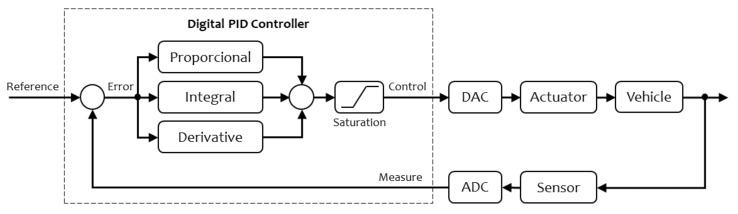
Closed-Loop Control System with a digital PID controller.

In the Steer-By-Wire and Throttle-By-Wire systems, a digital PID controller is implemented, as shown in Figure 22. The functions executed by the microcontroller are shown inside the square with a dashed line. The DAC and ADC are the devices that provide a connection between the microcontroller and the system. These two modules allow the analogue signals of the controlled system to be translated into digital numbers used by the microcontroller and vice versa.

The well known equations to implement the PID controller are as follows:

(4) Error=Reference−Measure

(5) $$Proportional=Error\cdot K_{P}$$

(6) $$Integral=Integral+Error\cdot K_{I}\cdot \Delta t$$

(7) $$Derivative=\left(Error-Error_{Previous}\right)\cdot \frac{K_{D}}{\Delta t}$$

(8) Control=Proportional+Integral+Derivative

The proportional parameter is calculated by multiplying the subtraction between the reference and the measure of the current state of the system, called error, by a constant of proportionality $\left(K_{P}\right)$. The proportional component can produce a significant error in steady state.

The integral component is calculated from the sum of all errors and the subsequent multiplication by an integral constant $\left(K_{I}\right)$. With the integral component, it is possible to reduce or eliminate the error in steady state produced by the proportional constant.

Finally, the derivative parameter is calculated from the subtraction of the current error minus the error in the previous instant, multiplied by a derivative constant $\left(K_{D}\right)$. This component introduces a corrective action that depends on the speed at which the error varies. The disadvantage of this component is that it amplifies the noise signals and can cause a saturation effect on the actuator.

The PID controllers tuning were performed empirically, firstly, adjusting the transient response by adjusting the proportional constant. Then, the error in steady state was decreased, increasing the integral constant. Finally, the derivative constant was added, but it affected the transitory worsening the system response. Consequently, it was eliminated.

PID is applied with a period (Δt) of 100 ms. Therefore, the sampling frequency of the sensors and actuators is 10 Hz. In the case of the Steer-By-Wire, an analogue signal is sent to the DC motor driver that operates the steering column. In the Throttle-By-Wire module, the control signal is sent to the AC traction motor controller, using the I2C bus and the external DAC.

### 4.6. ROS Module

This module has been implemented using the Raspberry Pi 3 model B, due to the need to select an embedded system able to support ROS with low consumption and low cost.

The main function of this node, which is executed every 100 ms, is to transform the linear speed and curvature setpoints into steering wheel turning angle and drive wheels turning speed variables using the vehicle’s kinematics. To do that, we use the Ackermann steering geometry [52].

**Figure 23:**
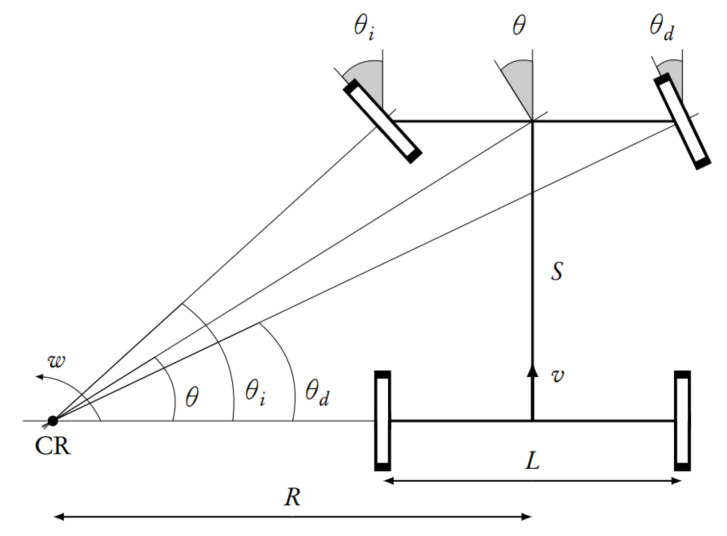
Ackermann steering geometry.

The vehicle has four wheels distributed on two axis, as shown in Figure 23. The driving and steering wheels are in the front, which turn thanks to the steering column and are powered by an AC induction motor. The rear wheels are kept straight and turn freely.

The angular velocity of the vehicle, in rad/s, is calculated by using the following equation:

(9) $$\omega =\dot{\theta }=\frac{\nu }{R}=\nu \cdot \kappa$$

where θ is the angle that a wheel located in the centre of the front axis would have (rad), ν is the vehicle’s linear speed that corresponds to the centre point of the rear axis (m/s), *R* is the turning radius (m) and κ is the curvature that is equal to the inverse of the turning radius ($m^{-1}$).

To calculate the turning angle of the front imaginary wheel, placed in the middle between the two real wheels, the right-angled triangle formed by the centre point of the front axis, the centre of rotation and the centre point of the rear axle is used, according to the following equation:

(10) $$tan\left(\theta \right)=\frac{S}{R}$$

where *S* is the wheelbase (m).

In the following, the angles of the drive wheels are calculated, as can be seen in Figure 23. The turning angle for these wheels are not the same. Right triangle formed by the centre of the wheel, the centre of the rear wheel on the same side and the centre of rotation are used. Since the lengths of both legs are known, one of them is the wheelbase (S) and the other the turning radius of the centre point of the rear axis, adding or subtracting half the length of the axis (L), the following equations are obtained:

(11) $$tan\left(\theta_{i}\right)=\frac{S}{R-\frac{L}{2}}$$

(12) $$tan\left(\theta_{d}\right)=\frac{S}{R+\frac{L}{2}}$$

In this context, a negative turn angle indicates a right turn. Remember that the turning radius R remains the centre of the vehicle’s rear axis. In this case, what is found is the turning angle of the steering wheel, which is proportional to the displacement that occurs in the steering rack. The ratio between the turning angle of the steering wheel (α) and the turning angle of the wheel (θ) is 40 and was empirically calculated in the vehicle. Therefore, the turning angle of the steering wheel can be obtained in radians, applying the following equation:

(13) $$\alpha =40\cdot \theta =40\cdot tan^{-1}\left(\frac{S}{R}\right)=40\cdot tan^{-1}\left(\kappa \cdot S\right)=40\cdot tan^{-1}\left(\frac{\omega \cdot S}{\nu }\right)$$

Finally, we calculate the angular speed, the front wheels must turn ($\omega_{w}$) in rad/s, knowing its radius (r) in metres and the required linear speed in the front wheels ($\nu_{w}$) in m/s:

(14) $$\omega_{w}=\frac{\nu_{w}}{r}=\frac{\nu }{cos(\theta )\cdot r}$$

Most measurement errors can be described by a random, Gaussian error process [53]. Assuming that the errors are not correlated, the sensitivity of the Steer-By-Wire and the Throttle-By-Wire to measurement errors are:

(15) $$\Delta \kappa^{2}={\left|\frac{\partial \kappa }{\partial \alpha }\right|}^{2}\cdot \Delta \alpha^{2}+{\left|\frac{\partial \kappa }{\partial S}\right|}^{2}\cdot \Delta S^{2}$$

(16) $$\Delta \nu^{2}={\left|\frac{\partial \nu }{\partial \alpha }\right|}^{2}\cdot \Delta \alpha^{2}+{\left|\frac{\partial \nu }{\partial \omega_{w}}\right|}^{2}\cdot \Delta \omega_{w}^{2}+{\left|\frac{\partial \nu }{\partial r}\right|}^{2}\cdot \Delta r^{2}$$

Therefore, the accuracy of the Drive-By-Wire system depends on the error in the measurement of the sensors ($\Delta \alpha ,\Delta \omega_{w}$) and the error in the measurement of the vehicle dimensions (ΔS,Δr). Using Equations (13) and (14), we obtain:

(17) $$\Delta \kappa^{2}={\left|sec^{2}\left(\frac{\alpha }{40}\right)\right|}^{2}\cdot {\left(\frac{1}{S\cdot 40}\right)}^{2}\cdot \Delta \alpha^{2}+{\left|tan\left(\frac{\alpha }{40}\right)\right|}^{2}\cdot \frac{1}{S^{4}}\cdot \Delta S^{2}$$

(18) $$\Delta \nu^{2}={\left|sin\left(\frac{\alpha }{40}\right)\right|}^{2}\cdot {\left(\frac{r}{\omega_{w}\cdot 40}\right)}^{2}\cdot \Delta \alpha^{2}+{\left|cos\left(\frac{\alpha }{40}\right)\right|}^{2}\cdot \frac{r^{2}}{\omega_{w}^{4}}\cdot \Delta \omega_{w}^{2}+{\left|cos\left(\frac{\alpha }{40}\right)\right|}^{2}\cdot \frac{1}{\omega_{w}^{2}}\cdot \Delta r^{2}$$

Therefore, the maximum errors will be obtained at the maximum turning points and at low speeds, for example a roundabout. For a low speed of 1 m/s, knowing the dimensions of the vehicle (S = 1.31 m, r = 0.28 m) and that the steering column rack allows a steering wheel turning range (α) of ±9.25 rad, the maximum errors are obtained:

(19) $$\Delta \kappa_{max}^{2}\approx \frac{1}{40^{2}\cdot S^{2}}\cdot \Delta \alpha^{2}+\frac{1}{20\cdot S^{4}}\cdot \Delta S^{2}=0.364\cdot 10^{-3}\cdot \Delta \alpha^{2}+29.1\cdot 10^{-3}\cdot \Delta S^{2}$$

(20) $$\Delta \nu_{max}^{2}\approx \frac{r^{2}}{20\cdot 40^{2}\cdot \omega_{w}^{2}}\cdot \Delta \alpha^{2}+\frac{r^{2}}{\omega_{w}^{4}}\cdot \Delta \omega_{w}^{2}+\frac{1}{\omega_{w}^{2}}\cdot \Delta r^{2}=\left(1.8\cdot 10^{-4}\cdot \Delta \alpha^{2}+5.8\cdot \Delta \omega_{w}^{2}+74.6\cdot \Delta r^{2}\right)\cdot 10^{-3}$$

The use of radians forces calculations with floating point data types. To increase the calculation speed, we decided to change the units to degrees. This allows the Olimexino-STM32 development board to perform all calculations with integer data types and reduce computation time.

**Figure 24:**
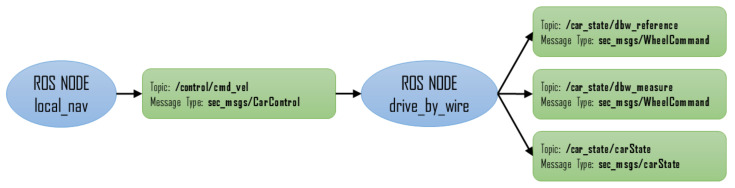
ROS Drive-By-Wire node diagram.

These setpoints are sent to the development board through a USB serial port. After sending the setpoints, the development board returns a response message with the vehicle’s current state. Based on these data, the vehicle’s variables are updated and published in three different topics. Figure 24 shows the operating diagram.

After receiving the response from the Olimexino-STM32, the value of the setpoints transformed to steering wheel turning angle and drive wheels turning speed are published in the topic /car_state/dbw_reference. In the topic /car_state/dbw_measure, the current value of the steering wheel turning angle and the drive wheels turning speed are published too. Finally, in the topic car_state/carState, the current states of the driving mode and the vehicle’s actuators are published.

As a security mechanism, the ROS setAutomaticMode service has been implemented, which is requested by the navigation system to the Drive-By-Wire system to set the autonomous driving mode. The operating scheme is shown in Figure 17. The vehicle starts in Manual mode. Pushing the Auto/Manual button on the control panel will set the Ready mode; this is the hardware activation mechanism.

In this mode, the system is prepared for autonomous driving as far as hardware elements are concerned, but the Drive-By-Wire ROS node sends null setpoints for safety. Therefore, the vehicle will remain stationary and with the steering wheel in the zero position. In this mode, the software activation mechanism must be activated. For this, the ROS service request must be sent to switch to Automatic mode, where the setpoints sent by the navigation system are sent to the Drive-By-Wire board and the vehicle follows these instructions accordingly.

If at any moment the driver activates any of the vehicle’s actuators (brake pedal, throttle pedal and steering wheel), the system will switch to Manual driving mode.

## 5. Experimental Navigation Results

The Drive-By-Wire system was tested and validated, during the development of the Techs4AgeCar project, as a part of the whole navigation architecture, in numerous use cases around the University Campus in a controlled way.

Hereafter, we present some tests to validate the Drive-By-Wire system. First, the step response of the Steer-By-Wire system and Throttle-By-Wire system is analysed. Second, the system response to the setpoints sent by the Control module is shown, when tracking a path defined by the Planner with the Perception and Decision modules disabled. Third, a test was carried out to validate the Manual/Automatic Switching module, where a trajectory was followed but, in this case, switching to manual driving in different sections of the route.

Finally, the data collected from the validation tests of the navigation systems that have been carried out in the last two years were analysed. From these data, the failures that occurred in these tests due to Drive-By-Wire were extracted and a study of the reliability of the system was made.

### 5.1. Step Response

**Figure 25:**
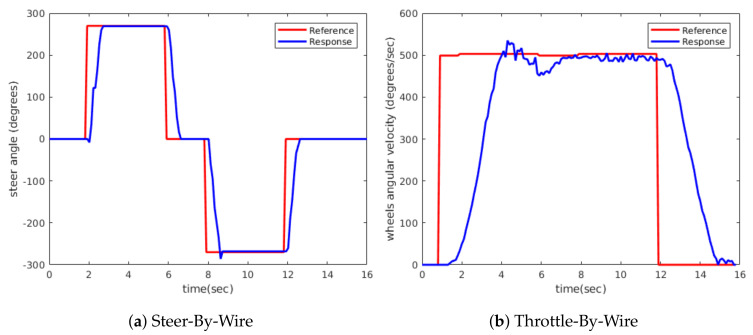
Drive-By-Wire step response for two people.

**Figure 26:**
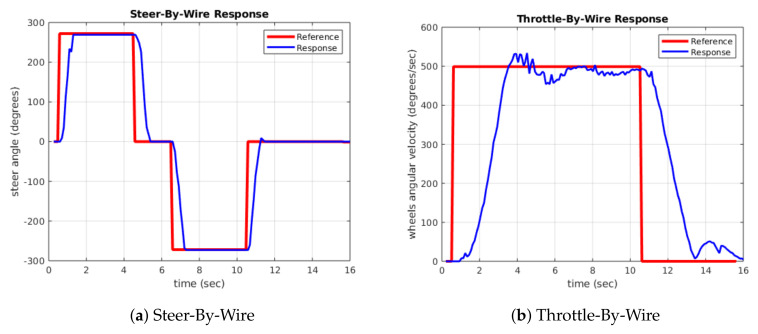
Drive-By-Wire step response for four people.

The transient response of the system was analysed for different loading conditions. First, the PID controller was tuned with a load of two people until the desired behaviour, as shown in Figure 25, was obtained. The response of the system was then analysed for different load conditions and it was observed that the response is adequate for the different load conditions required, as in the case of four people shown in Figure 26.

The transient response to a step input of the Steer-By-Wire corresponds to Figure 25a and Figure 26a, in which an underdamped response can be observed for both rotational directions. The Deadtime for both responses is 100 ms. The Rise Time is approximately 650 ms when it reaches 90% of its Steady-State Response. The Setting Time is 799 ms, the Maximum Peak Overshoot is 3.92% and the Steady-State Error is ±4°, which supposes a ±0.1° error in the drive wheels turning angle.

The Throttle-By-Wire also has an underdamped response, as seen in Figure 25b and Figure 26b. In this case, a slow response is sought, to obtain a smooth and fluid driving. The Deadtime is 320 ms. The Rise Time is 2.20 s when it reaches 90% of its Steady-State Response and the Setting Time is 4.80 s. For a load of four people, these times increase to 2.39 and 5.45 s, respectively. In both cases, the Maximum Peak Overshoot is 4.73% and the Steady-State Error is ±20°/s, equivalent to ±0.36 km/h error in the vehicle’s linear speed.

The response and the error obtained for the two systems are good enough to be used in an electric vehicle’s autonomous navigation system.

### 5.2. Path Tracking

**Figure 27:**
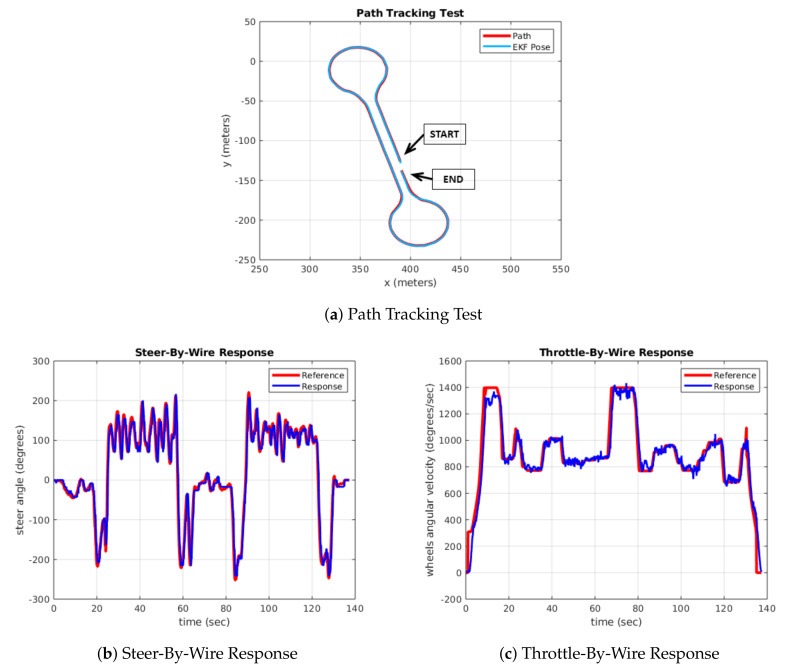
Path tracking test of the Drive-By-Wire response.

Figure 27 shows the results obtained by following a path defined by the Global Planner, in which the Control Module sends the necessary setpoints to follow the path. The Perception and Decision-Making Modules are disabled because, at this level of validation, they do not provide relevant information.

The reference path and the performed path are shown in Figure 27a. The vehicle travelled a distance of approximately 800 m, where it performed two roundabouts and finally it returned to the starting point. The maximum linear speed configured in the Control Module is 25 km/h, which is equivalent to a wheel turning speed of 1387°/s. The time response of the Steer-By-Wire system is depicted in Figure 27b, and the Throttle-By-Wire response in Figure 27c.

As can be seen, the Drive-By-Wire system is capable of responding adequately to the commands sent by the controller. As a result of the good communication and functioning of these two modules, the vehicle is able to follow the trajectory, obtaining a Root Mean Square Error (RMSE) of 0.347 m and a maximum error of 0.863 m in the roundabout area.

### 5.3. Manual/Automatic Switching

**Figure 28:**
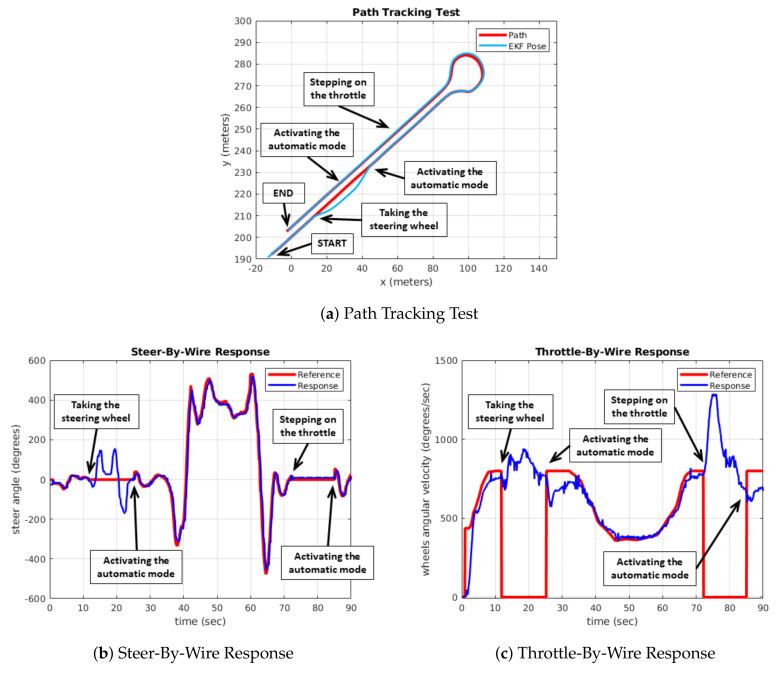
Manual/Automatic switching test.

Figure 28 shows the results obtained in the test to validate the Manual/Automatic switching module, where the same trajectory of the previous test is followed, but in this case the switch to manual driving is carried out in different positions of the route.

The test starts in Automatic mode. After 12 s, the steering wheel is actuated and the vehicle change to Manual mode. The driver takes the control for 15 s and then Automatic mode is activated again. At 73 s, the throttle pedal is actuated and the vehicle returns to Manual mode for 15 s, and finally it is switched back to Automatic mode until the end of the test.

As can be seen in Figure 28b,c, transition between modes is smooth. When it is switched to Manual mode, the setpoints sent to the Drive-By-Wire module are null. In these cases, the answer is given by the manual activation of the throttle and steering wheel turning signals. As soon as the Automatic mode is switched back to, the control module generates the corresponding setpoints and the Drive-By-Wire system conveniently follows it. Control transitions are shown on the path (Figure 28). As can be seen, in the first manual control takeover by turning the steering wheel, the driver performs a manual lane change. When control is automatic again, the system returns to the predefined trajectory. The second commutation is carried out by operating the throttle, which causes a manual increase in linear speed on the straight, until it is returned to automatic control and the vehicle returns to the reference speed.

### 5.4. Reliability Analysis

The data collected from the validation tests of the navigation systems, which have been carried out in the last two years, were analysed. From these data, the failures that occurred in these tests due to Drive-By-Wire were extracted and a reliability study of the system was made.

In total, 812 h of tests were extracted, in which the system was in operation for 788 h. The rest of the time the system was down due to 29 Drive-By-Wire system failures. All the formulas used for the reliability analysis were taken from the IEEE standards presented in [54].

The Mean Time Between Failures (MTBF) is calculated using Equation (21). This metric provides a rough estimate of how long you can expect to use the Drive-By-Wire without encountering faults. Reliability (R) is the probability that the system will function properly for a required time. Another metric used in this analysis is the Failure Rate (λ), which is simply the inverse of the MTBF. Availability (A) is calculated using Equation (25), where the Mean Time To Repair (MTTR) is defined in Equation (24).

(21) $$MTBF=\frac{N{}^{\circ}\mathrm{hours}\mathrm{of}\mathrm{operation}}{N{}^{\circ}\mathrm{failures}}=\frac{788}{29}=27.17\mathrm{hours}$$

(22) $$\lambda =\frac{1}{MTBF}=\frac{1}{27.17}=0.0368\mathrm{failures}/\mathrm{hour}$$

(23) $$R\left(t\right)=e^{-\lambda \cdot t}=e^{-0.0368\cdot t}$$

(24) $$MTTR=\frac{N{}^{\circ}\mathrm{hours}\mathrm{spent}\mathrm{repairing}}{N{}^{\circ}\mathrm{failures}}=\frac{812-788}{29}=0.8275\mathrm{hours}$$

(25) $$A=\frac{MTBF}{MTBF+MTTR}\cdot 100=\frac{27.17}{27.17+0.8275}\cdot 100=97.04\%$$

The results show that the Reliability of the Drive-By-Wire system is high, with an MTBF of 27 h and an Availability of 97.04%.

## 6. Conclusions

This paper presents the development process of a robust and ROS-based Drive-By-Wire system for an autonomous electric vehicle designed for the Techs4AgeCar project.

Firstly, a study of current technology and different available solutions was carried out. Given the shortage of existing open source proposals and their high prices, we decided to automate an open source electric chassis from scratch. We started by developing the Steer-By-Wire system, replacing the mechanical steering column of the vehicle for an electric one operated thanks to a DC motor. After that, we tackled the Throttle-By-Wire implementation, which presented less complexity than the previous system, as it was an electric vehicle. At this point, we observed that the braking achieved with the regenerative brake of the electric motor was sufficient for our first autonomous navigation validation tests and decided not to automate at the moment this system.

Once the automation of the vehicle was achieved, we performed the corresponding adjustments to obtain an efficient and robust system. During these tests, we identified the need to design a manual/automatic switching system that allowed the driver activating the autonomous driving and safely taking control of the vehicle at any time.

After that, we developed the communication interface with the navigation system, based in ROS, over a Raspberry Pi.

We performed some validation tests for our Drive-By-Wire system as a part of our whole autonomous navigation architecture, showing the good working of our proposal. Firstly, the step response of the Steer-By-Wire system and Throttle-By-Wire system was analysed. Secondly, the system response to the setpoints sent by the Control module was shown, when tracking a path defined by the Planner with the Perception and Decision modules disabled. Thirdly, a test was carried out to validate the Manual/Automatic Switching module, where the same trajectory of the previous test was followed, but, in that case, switching to manual driving in different sections of the route.

Finally, the data collected from the validation tests of the navigation systems, which were carried out in the last two years, were analysed. From these data, the failures that occurred in these tests due to Drive-By-Wire were extracted and a study of the reliability of the system was made.

The results prove that the Drive-By-Wire system developed has the behaviour and the necessary requirements to automate an electric vehicle. Once implemented within an autonomous navigation architecture based on ROS, the results show that the system responded adequately to the commands sent by the high level. This allows the autonomous vehicle to follow a trajectory safely, since the system has an autonomous driving mode switching module, which allows taking control of the vehicle at any time. In addition, after 812 h of testing, it was proven that it is a robust Drive-By-Wire system, with high reliability.

Currently, there are very few Drive-By-Wire projects based on ROS and, in the state of the art, none of them is Open-Source. The developed system is the basis for the validation and implementation of new autonomous navigation techniques developed within the group in a real vehicle. In addition, it serves as a platform for capturing perception and control data, which will allow the creation of databases and the development of autonomous driving algorithms based on Deep-Learning.

Future work includes the development of the Brake-By-Wire system, a remote control interface for the vehicle based on ROS and the implementation of a more robust and safer communication bus (CAN, FlexRay, etc.) between control units of the vehicle.

## Figures and Tables

**Table 1:** Technical specifications of the Techs4AgeCar’s vehicle.

Parameter	Value	Parameter	Value
**Length**	3030 mm	**Max. Climb**	25%
**Width**	1488 mm	**Turn Radius**	5 m
**Height**	1380 mm	**Max. Steering Angle**	±20°
**Weight (Batteries incl.)**	730 kg	**Max. Motor Power**	29.5 kW @ 2500 rpm
**Batteries Weight**	140 kg	**Max. Motor Torque**	128 N·m @ 500 rpm
**Wheelbase**	2360 mm	**Max. Motor RPM**	5500 rpm
**Track Width**	1315 mm	**Rated Voltage**	80 Vac
**Top Speed**	120 km/h	**Max. Current**	400 A
**Nominal Range**	100 km	**Max. Power Factor**	0.97
**Tires**	175/55R15	**Reduction Gearbox Ratio**	5.8:1
